# Determining the learning curve for percutaneous endoscopic lumbar interbody fusion for lumbar degenerative diseases

**DOI:** 10.1186/s13018-023-03682-z

**Published:** 2023-03-12

**Authors:** Tingxiao Zhao, Zhanqiu Dai, Jun Zhang, Yazeng Huang, Haiyu Shao

**Affiliations:** 1 Center for Plastic & Reconstructive Surgery, Department of Orthopedics, Zhejiang Provincial People’s Hospital (Affiliated People’s Hospital, Hangzhou Medical College), Shangtang Road 158#, Hangzhou, 310014 Zhejiang China; 2 Bengbu Medical College, Bengbu, Anhui China

**Keywords:** Learning curve, Minimally invasive surgery, Percutaneous endoscopic lumbar interbody fusion, PELIF

## Abstract

**Purpose:**

Percutaneous endoscopic lumbar interbody fusion (PELIF) is one of the least invasive procedures for lumbar degenerative disorders (LDD). There is limited knowledge of the learning curve for PELIF.

**Methods:**

A total of 93 consecutive patients who underwent PELIF performed by a single spine surgeon for LDD failed with conservative treatment were retrospectively reviewed. The case series was split into three groups based on timing: A (earliest third of patients); B (middle third of patients); and C (latest third of patients). The following were also recorded: operating time, X-ray exposure time, complications, radiologic fusion rates, pre- and postoperative patient-reported outcome measures (PROMs) scores (visual analogue scale (VAS) for back pain, VAS for leg pain, Japanese Orthopaedic Association, Oswestry Disability Index and MacNab criteria), length of hospital stay, and need for revision surgeries. A learning curve was then developed by a logarithmic curve-fit regression analysis.

**Results:**

The operative time gradually decreased over time, and an asymptote was reached after about 25 cases. Compared with group B or C, group A had significantly longer operative time, significantly longer length of hospital stay, needed significantly more x-ray exposure time. Though not significantly different, there are fewer complications and revision surgeries over time. There is no significant difference over time in PROMs scores except for the VAS back scores.

**Conclusions:**

PELIF is an alternative for minimal invasive surgery for LDD, PELIF presents a learning curve to the practicing spine surgeon with regard to operative time, x-ray exposure time, length of hospital stay, clinical PROMs and radiographic outcomes and complications. The presented PELIF learning curve provided valuable insight to surgeons interested in performing this surgery.

## Introduction

When nonoperative management fails in treating patients with lumbar generative disorders (LDD) (e.g., lumbar instability, stenosis, spondylolisthesis), lumbar interbody fusion (LIF) is commonly performed [1–6]. Indeed, since Briggs and Milligan first described a posterior lumbar interbody fusion (PLIF) in 1944, this operative approach has been widely accepted globally [7]. Although conventional PLIF may achieve decompression of critical neural structures and restore an acceptable level of spine, the use of PLIF has been limited due to its risk of perioperative complications caused by significant destruction to the posterior muscle-ligament complex [8–12]. In order to minimize injury risk to these soft tissues, various minimally invasive surgical (MIS) techniques, including anterior lumbar interbody fusion (ALIF), direct lateral lumbar interbody fusion (DLIF), oblique lumbar interbody fusion (OLIF), and MIS-transforaminal lumbar interbody fusion (MIS-TLIF), have been developed and have gradually gained popularity [13–16].

In recent years, with the development of surgical techniques and instruments, percutaneous endoscopic lumbar interbody fusion (PELIF) has been increasingly used to treat LDD [17–19]. According to research reports, PELIF has significant advantages. It can not only achieve decompression effect by removing the intervertebral disc, but also achieve endoscopic interbody fusion. During the operation of PELIF, the endoscope can be directly inserted into the intervertebral disc space. The cartilage endplate can be completely removed without damaging the bone endplate under a clear and visual image. Good preparation of the endplate can promote the intervertebral fusion [20]. In addition, PELIF can be performed under local or epidural anesthesia, which reduces the risk of anesthesia, which is of great significance to patients who cannot accept general anesthesia [21]. Compared with traditional open surgery, PELIF has less intraoperative bleeding, shorter hospital stay and significantly improved postoperative pain [22]. However, PELIF still has some limitations. Due to the complex anatomical structure of the intervertebral foramen, surgery often requires surgeons with rich experience in endoscopic decompression. However, PELIF is a challenging due to the limited operative working space available and high risk of potential complications; therefore, achieving the best clinical outcomes likely requires not only robust surgical knowledge but also operative experience [23].

Currently, the literature reports the learning curves of TLIF, DLIF, MIS-TLIF, and OLIF [24–27]. However, there is limited knowledge of the learning curve of PELIF. In this study, we aim to develop and evaluate the learning curve of PELIF.

## Methods

### Patient population

Following approval of the appropriate Institutional Review Board, we retrospectively reviewed cases of patients who underwent PELIF by a single spine surgeon (YZH). This surgeon has 15 years of attending spine surgeon experience. All surgeries were done between Oct. 2017 and Apr. 2020. All patients had a minimum of 12 months follow-up (mean 18.6 months, range from 12 to 30 months). Patients were excluded if they had severe osteoporosis, severe degenerative or congenital spinal deformity, greater than grade II spondylolisthesis (Meyerding grade III and IV), severe central spinal stenosis, intraspinal pathology, or infection. In addition, patients who received any surgical intervention at L5/S1 segment were excluded because of the obstruction from the iliac crest. All other patients were included.

Ultimately, a total of 93 consecutive cases meeting the inclusion criteria were analyzed. Ninety patients underwent only single-level surgery, and three patients underwent double-level PELIF. For all analyses, patients were divided sequentially into three groups: A (earliest third of patients); B (middle third of patients); and C (latest third of patients).

### Surgical technique

Each procedure was performed with the patient in the prone position on a radiolucent operating table. Local anesthesia supplemented with neuroleptic analgesia [Dexmedetomidine (1 μg/kg during 10 min for loading dose and 0.2–0.7 μg/kg/hr for maintenance dose)] was utilized during decompression. An epidural anesthesia (EA) tube is prepared preoperatively but is only used if patients complain of unbearable pain during bone removal, expandable cage implantation, and/or percutaneous pedicle screw placement.

More detailed information of the procedure for decompression and fusion is available in Authors previously published reports [22, 28].

### Clinical and radiologic evaluation

The following clinical data were collected: age (years), sex (man or woman), recorded diagnosis, fusion level (s), operating time (minutes), X-ray exposure time (minutes), perioperative complications, length of hospital stay (day), and clinical outcomes. The following patient-reported outcome measures (PROMs) were collected: visual analog scale (VAS) for back pain; VAS for leg pain; Japanese Orthopedic Association (JOA), Oswestry Disability Index (ODI), and MacNab criteria. All of the patients were followed up for at last one year. Plain radiographs and CT scans were performed and assessed by an independent radiologist and the surgeon who performed the surgery during follow-up. Definitive fusion was defined as visualized osseous continuity between the graft bone and vertebrae with no obvious zone between the graft bone/cages and vertebrae on CT scan, and < 3 mm translational motion and < 5° segmental movement on the flexion–extension lumbar radiographs [29].

### Statistical analysis

Descriptive statistics were calculated. Evaluations of operative time, x-ray exposure time, and length of hospital stay were conducted using logarithmic curve-fit regression analyses. Comparisons were performed using chi-square tests or Fisher’s exact tests, when appropriate, for categorical variables and Wilcoxon rank-sum tests for continuous variables.

All analyses were performed using SAS software, version 9.4 (SAS Institute, Cary, NC, USA). For all analyses, *p*-value < 0.05 was considered statistically significant.

## Results

### Demographic data

**Fig. 1:**
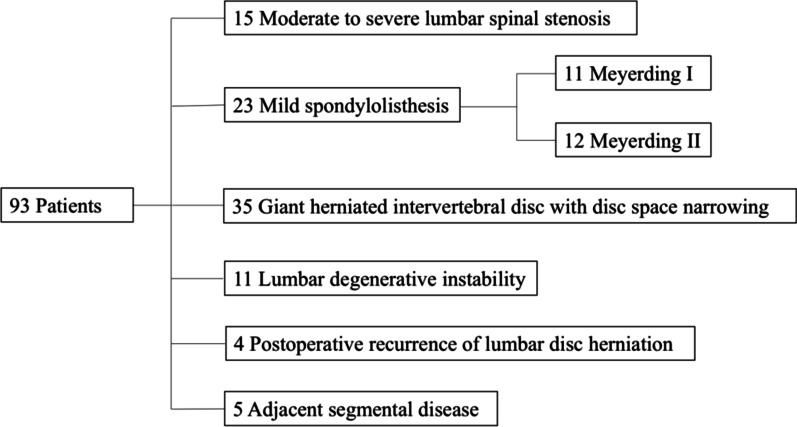
Schematic diagram summarizing the clinical diagnoses of ninety-three consecutive patients received PELIF

Of the 93 patients, a majority were women (*n* = 49 [53%]) and the average age was 67 years (range, 40–90 years) (Table 1). The causes of disease included moderate to severe stenosis for 15 patients, mild spondylolisthesis for 35 patients (Meyerding grade I and II), giant herniated intervertebral disc with intervertebral disc space narrowing for 35 patients, lumbar degenerative instability for 11 patients, postoperative recurrence of lumbar disc herniation for 4 patients, and adjacent segmental disease for 5 patients. The breakdown of diagnoses is shown in Fig. 1. Across the entire patient sample, the mean duration of symptom was 10.9 months. There was no significant difference in age, sex, mean duration of symptoms, or diagnosis, among the three groups, however, the level(s) of fusion were significantly different.

**Table 1:** Patient Demographics

Variables	Total	Group A	Group B	Group C	*p*-value
Patients, *n*	93	31	31	31	
Age (years)					0.50
Mean	66.7	68.2	67.4	64.5	
Range	40–90	43–83	42–90	40–89	
Sex (*n*)					0.81
Male	44	16	15	13	
Female	49	15	16	18	
Duration of symptoms (months)					0.92
Mean	10.9	10.4	11.2	11.3	
Range	3–24	4–18	3–24	4–22	
Diagnosis, *n* (%)					0.83
Moderate to severe lumbar spinal stenosis	15 (16.1%)	7 (22.6%)	4 (12.9%)	4 (12.9%)	
Mild spondylolisthesis (Grade I or II)	23 (24.7%)	6 (19.4%)	10 (32.3%)	7 (22.6%)	
Giant herniated intervertebral disc with disc space narrowing	35 (37.6%)	13 (41.9%)	11 (35.5%)	11(35.5%)	
Lumbar degenerative instability	11 (11.8%)	4 (12.9%)	3 (9.7%)	4 (12.9%)	
Postoperative recurrence of lumbar disc herniation	4 (4.3%)	1 (3.2%)	1 (3.2%)	2 (6.5%)	
Adjacent segmental disease	5 (5.4%)	0	2 (6.5%)	3 (9.7%)	
Levels Fused, *n* (%)					0.01
L2-3	9 (9.7%)	2 (6.5%)	4 (12.9%)	3 (9.7%)	
L3-4	22 (23.7%)	13 (41.9%)	2 (6.5%)	7 (22.6%)	
L4-5	59 (63.4%)	16 (51.6%)	25 (80.6%)	18 (58.1%)	
L2/3 + L3/4	1 (1.1%)	0	0	1 (3.2%)	
L3/4 + L4/5	2 (2.2%)	0	0	2 (6.5%)

### Operation time

**Fig. 2:**
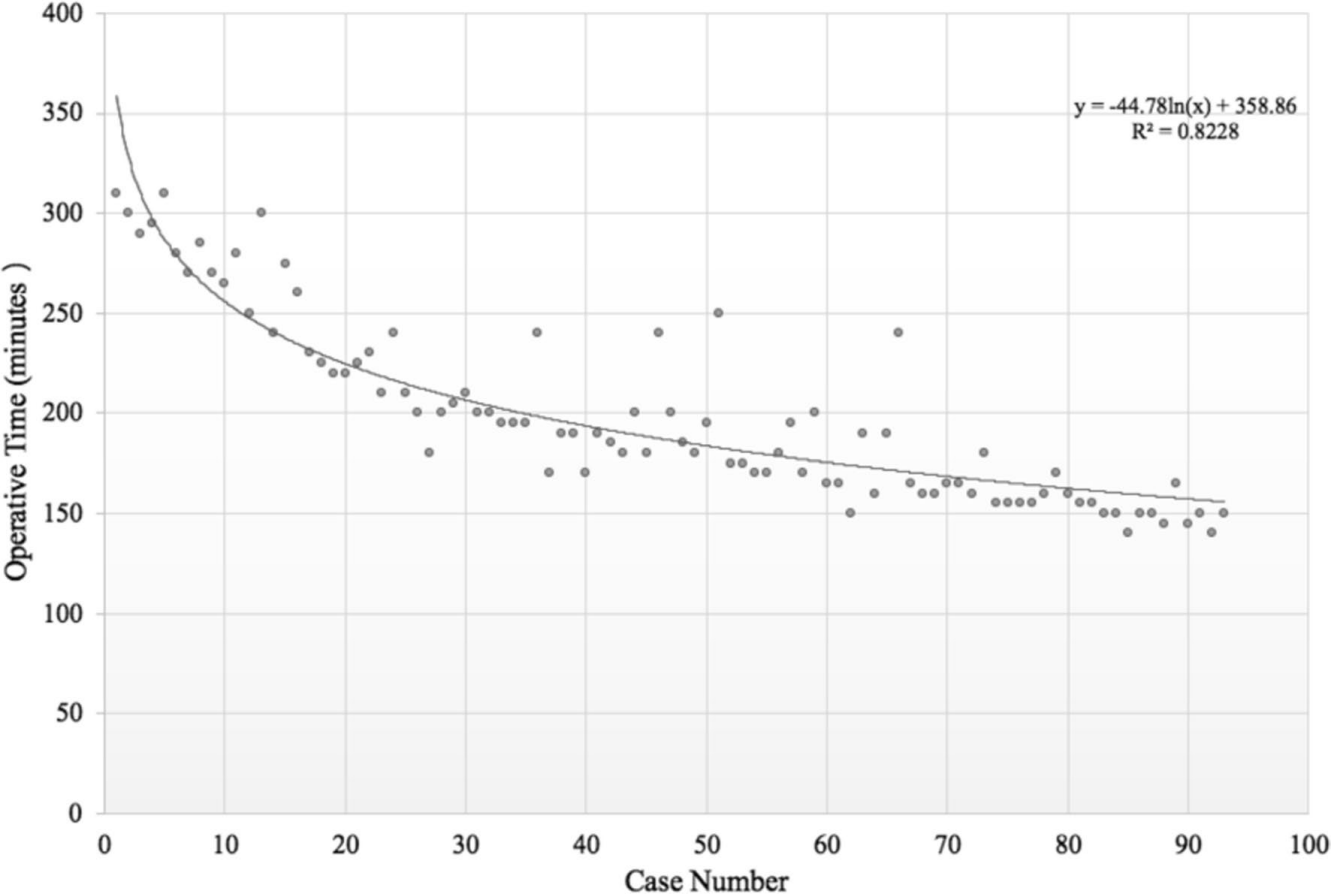
Learning curve of PELIF as shown by operative time

Operative time decreased significantly from 247.90 ± 38.18 min in group A to 188.55 ± 22.22 min in group B to 160.97 ± 18.95 min in group C (*p* < 0.001) (Table 2). Further, operative time decreased significantly as case number increased (p < 0.001; equation: *y* = − 44.78 ln(*x*) + 358.86, R^2^ = 0.348, where case number = *x* and operative time in minutes = *y*) (Fig. 2). The steady state, which is defined as the asymptote of the learning curve, was estimated to be achieved at around the 25^th^ case.

**Table 2:** Comparison of Clinical Outcomes

Clinical outcome variable	Group A (Mean ± SD)	Group B (Mean ± SD)	Group C (Mean ± SD)	*p*-value
Operative time (minutes)	247.90 ± 38.18	188.55 ± 22.22	160.97 ± 18.95	< 0.001
X-ray exposure time (seconds)	63.71 ± 4.26	51.84 ± 4.71	39.42 ± 4.51	< 0.001
Length of Hospital stay (days)	6.87 ± 0.72	6.61 ± 0.67	5.68 ± 0.65	< 0.001
Complications, *n* (%)	3 (9.6%)	1 (3.2%)	0	0.32
Need for revision surgery, *n* (%)	2 (6.5%)	1 (3.2%)	0	0.77

### X-ray exposure time

**Fig. 3:**
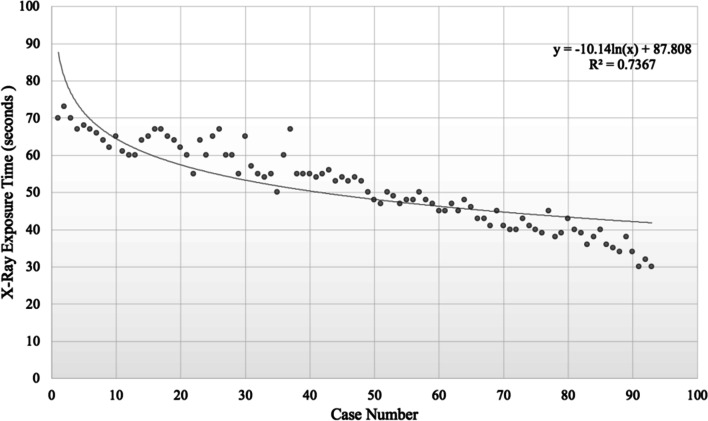
Learning curve of PELIF as shown by X-ray exposure time

X-ray exposure time significantly decreased from 63.71 ± 4.26 s in group A to 51.84 ± 4.71 s in group B to 39.42 ± 4.51 s in group C (*p* < 0.001) (Table 2). In addition, X-ray exposure time decreased significantly as case number increased (p < 0.001; equation: *y* = − 10.14 ln(*x*) + 87.808, R^2^ = 0.7367, where case number = *x* and X-ray exposure time in seconds = *y*) (Fig. 3). The steady state was estimated to be achieved at around the 30th case.

### Length of hospital stay

**Fig. 4:**
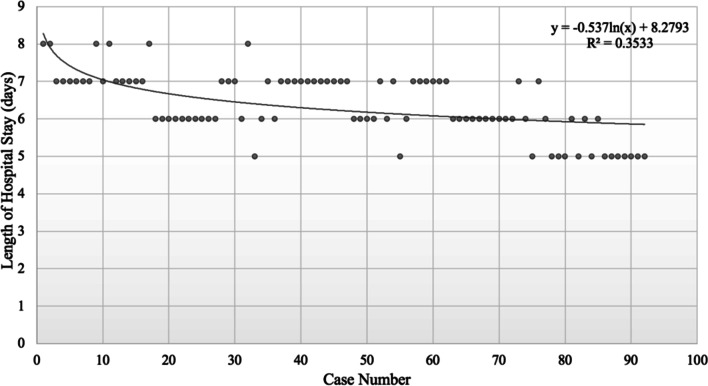
Learning curve of PELIF as shown by length of hospital stay

Length of hospital stay decreased significantly from 6.87 ± 0.72 days in group A to 6.61 ± 0.67 days in group B to 5.68 ± 0.65 days in group C (*p* < 0.001) (Table 2). However, length of hospital stay did not significantly decrease as case number increased (*p* = 0.431; equation: *y* = 0.1357 ln(*x*) + 5.8868, R^2^ = 0.0226, where case number = *x* and length of hospital stay in days = *y*) (Fig. 4). Because there was no significant association, the steady state was unable to be determined.

### Complications

There was no significant difference in perioperative complication rates among the three groups (*p* = 0.3187) (Table 2). Overall, however, a total of three, one, and zero patients had complications in groups A, B, and C, respectively. In group A, symptoms secondary to contralateral nerve root compression was found in two patients, which was caused by residual intervertebral disc. Ultimately, these symptoms completely released after PELD revision surgeries. The final complication in group A was a single patient found to have an infection. Following debridement without removal of instrumentation, this patient recovered fully. In group B, the one complication was secondary to a misplaced L5 pedicle screw. The patient exhibited severe radiculopathy after the index surgery, and the symptom resolved fully after the misplaced screw was removed. As briefly mentioned, it was notable that no complications were observed in group C.

### Clinical PROMs and radiographic outcomes

Among the three patient groups, there was no significant difference in VAS leg pain, JOA, or ODI scores; however, VAS back scores improved from group A to groups B and C (p = 0.0013) (Table 3). Further, no significant difference was detected in MacNab Criteria results (Table 4).

**Table 3:** Comparison of PROMs

PROM	Groups	Preoperative (Mean ± SD)	Postoperative (Mean ± SD)	Improvement (Mean ± SD)	p-value
VAS—Back	Group A	6.19 ± 0.54	2.42 ± 0.50	3.77 ± 0.56	0.001
	Group B	6.68 ± 0.48	2.32 ± 0.48	4.35 ± 0.66	
	Group C	6.48 ± 0.51	2.19 ± 0.40	4.29 ± 0.74	
VAS—Leg	Group A	8.32 ± 0.65	2.39 ± 0.50	5.94 ± 0.85	0.06
	Group B	8.45 ± 0.57	2.23 ± 0.43	6.23 ± 0.76	
	Group C	8.65 ± 0.49	2.23 ± 0.43	6.42 ± 0.62	
JOA	Group A	14.19 ± 2.09	26.19 ± 2.15	− 12 ± 2.96	0.16
	Group B	14.45 ± 2.42	26.32 ± 1.14	− 11.87 ± 2.50	
	Group C	14.84 ± 2.22	26.13 ± 1.02	− 11.29 ± 2.19	
ODI	Group A	67.87 ± 7.57	11.29 ± 3.64	56.59 ± 7.17	0.74
	Group B	67.81 ± 6.88	11.48 ± 3.78	56.32 ± 6.95	
	Group C	69 ± 6.47	11.23 ± 3.73	57.77 ± 7.18	

VAS: Visual analog scale; JOA: Japanese Orthopedic Association; ODI: Oswestry Disability Index

**Table 4:** Comparison of MacNab Criteria

MacNab criteria outcome	Group A, *n* (%)	Group B, *n* (%)	Group C, *n* (%)	*p* value
Excellent	18 (58%)	24 (77.4%)	24 (77.4%)	0.20
Good	11 (35.5%)	7 (22.6%)	7 (22.6%)	
Fair	0	0	0	
Poor	2 (6.5%)	0	0	

Assessment of radiographic fusion was based on modified Bridwell criteria, with grades I or II indicating “fused” and grades III or IV indicated “not fused”. Overall, the fusion rates of groups A, B, and C were 83.9%, 80.6%, and 87.1%, respectively. There was no significant difference in fusion rates among the three groups (*p* = 0.6905) (Table 5).

**Table 5:** Radiological Fusion Rate of PELIF Based on Modified Bridwell Classification

Bridwell grades	Group A, *n* (%)		Group B, *n* (%)		Group C, *n* (%)		*p*-value
I	12	26 (83.9%)	17	25 (80.6%)	1	27 (87.1%)	0.788
II	14		8		10		
III	5	5 (16.1%)	6	6 (19.4%)	4	4 (12.9%)	
IV	0		0		0		

## Discussion

Over the most recent decades, the number of spine patients requiring LIF has steadily increased [30, 31]. Although conventional PLIF can decompress neural structures and stabilize the spine, it has disadvantages, such as large trauma, high intraoperative blood loss, and destruction of normal anatomical structures, resulting in postoperative low back pain and prolonged recovery time [8–12]. In an effort to reduce the potential complications related with open surgery, the trend for LDD treatment has shifted to MIS procedures, including ALIF, DLIF, OLIF, MIS-TLIF, and PELIF. Notably, PELIF has evolved rapidly because it allows for adequate visualization, minimal muscle disruption, and good clinical and radiologic outcomes [24, 28, 32, 33]. However, as with any novel surgical technique, there is a learning curve. In the present study, we demonstrate that operative time and X-ray exposure time are optimized around the 25th and 30th procedures, respectively. This is accomplished with no change in complication rates or worsening of clinical outcomes and PROMs.

In addition to ensuring clinical outcomes are equivalent or better than those achieved using traditional surgical techniques, it is important to understand the learning curve of novel surgical procedures as well. Such knowledge is essential to understanding the caseload needed before an appropriate level of mastery can be developed. Further, this information is crucial for teaching and evaluation purposes. Our study indicated that there is a "long" learning curve for PELIF initially, but it can be overcome, as expected, with a greater number of cases. By gradually accumulating experience with PELIF, spine surgeons can appropriately adapt to the high technical demands of the procedure, which are required because the approach has a small operative window and provides a limited field of view [34, 35].

In addition to the demonstrated learning curve, it is important to report on complications and clinical outcomes. This is especially true as complications are the major concern of surgeons considering PELIF as a surgical approach. In our study, only four patients (4.3% of 93 patients) had complications. Among them, two cases had symptoms consistent with contralateral nerve root compression, while another case had a L5 pedicle screw mis-placement. We believe these complications may be reflective of surgeon inexperience, as no similar complications were observed in group C. In addition, the overall infection rate in our sample was 1.1% (1 of 93 patients), which is notably lower than other rates reported in LIF surgery literature [8–15]. Importantly, no severe perioperative complications, such as dural tears, intervertebral space infection, or implant loosening, were observed in our patient sample. Lastly, patients improved across all clinical outcomes and PROMs regardless of when they received surgery. These findings demonstrate that PELIF is a promising alternative MIS procedure for patients with LDD.

In addition, clinical efficacy and fusion rate are the top-rated concerns for a novel fusion technique. Previous studies on PELIF reported good or excellent relief of radiculopathy and low-back pain, and satisfactory interbody fusion rate was obtained [28]. Assessment of radiographic fusion was based on modified Bridwell criteria, with grades I or II indicating “fused” and grades III or IV indicated “not fused”. Overall, the fusion rate of all patients was 83.9%. Compared with previous studies, our series showed relatively higher fusion rate which could be attribute to the modifications we made. First, endoscopic visualization could provide double check combined with the surgeon’s experience for endplate preparation. Second, we used autogenous iliac bone as bone graft for all of the patients, which is the “gold-standard” for bone graft. Finally, all patients were instrumented with percutaneous pedicle screw-rod system, which could increase the stability of target level. All the above three modifications could ensure the higher fusion rate than the published studies of PELIF.

Importantly, because there is a learning curve in PELIF, as there are for all surgical procedures, younger spine surgeons learning this technique should be monitored closely. Further, even more experienced spine surgeons attempting this novel approach should seek guidance from those with more experience specifically with PELIF. If possible, as surgical simulation models improve, it may be safest for surgeons to practice PELIF using them. More research would then be required to determine the PELIF learning curve using simulation models and how the results translate to true surgeries. Ultimately, simulation may offer the safest approach to learning PELIF. Our study does not account for whether spine surgeons must operate a certain amount in order to not “fall down” the learning curve. This is an important consideration, as prior research has demonstrated that surgeons who perform a certain procedure more often (i.e., higher volume) have better outcomes [36]. Lastly, there are several factors that may impact the learning curve for a given procedure. However, by only including patients operated on by a single spine surgeon, we aimed to reduce any confounders as much as possible.

This study has several limitations. First, operations performed by a single spine surgeon at a single hospital limit the generalizability of the learning curve. Second, the retrospective nature of this study may lead to bias. Third, because the overall complication rate of this study is low, this study may not have sufficient evidence to determine the difference between early and late cohorts.

## Conclusions

Using a 93 sample from a single spine surgeon, we developed and evaluated the PELIF learning curve. As a surgeon performs more operations, decreases in operation time and X-ray exposure time are observed. We found that operation time and X-ray exposure time indicators appear to be optimized after performing 25–30 surgeries. However, no significant difference in hospital length of stay, complication, clinical PROMs and radiographic outcomes were demonstrated with increased surgeon experience. In summary, the results of this study also suggest that PELIF remains a safe and effective treatment option for LDD, despite the high early operation time and X-ray exposure time. Besides, future work can build upon our findings to determine the possible variation in PELIF learning curve by surgeon experience.

## Abbreviations

PELIF: Percutaneous endoscopic lumbar interbody fusion
LDD: Lumbar degenerative disorders
PROMs: Patient-reported outcome measures
VAS: Visual analogue scale
JOA: Japanese Orthopaedic Association
ODI: Oswestry Disability Index
LIF: Lumbar interbody fusion
PLIF: Posterior lumbar interbody fusion
MIS: Minimally invasive surgery
ALIF: Anterior lumbar interbody fusion
DLIF: Direct lateral lumbar interbody fusion
OLIF: Oblique lumbar interbody fusion
MIS-TLIF: Minimally invasive surgery-transforaminal lumbar interbody fusion
PELD: Percutaneous endoscopic lumbar discectomy
EA: Epidural anesthesia
